# Supplementation with *Lactiplantibacillus brevis* GKEX Combined with Resistance Exercise Training Improves Muscle Mass, Strength Performance, and Body Fat Condition in Healthy Humans

**DOI:** 10.3390/foods13071030

**Published:** 2024-03-27

**Authors:** Mon-Chien Lee, Yi-Ju Hsu, Chin-Shan Ho, You-Shan Tsai, Chin-Chu Chen, Chi-Chang Huang

**Affiliations:** 1 Graduate Institute of Sports Science, National Taiwan Sport University, Taoyuan 333325, Taiwan; 1061304@ntsu.edu.tw (M.-C.L.); ruby780202@ntsu.edu.tw (Y.-J.H.); kilmur23@ntsu.edu.tw (C.-S.H.); 2 Center for General Education, Taipei Medical University, Taipei 110301, Taiwan; 3 Biotech Research Institute, Grape King Bio Ltd., Taoyuan 325002, Taiwan; youshan.tsai@grapeking.com.tw (Y.-S.T.); gkbioeng@grapeking.com.tw (C.-C.C.); 4 Institute of Food Science and Technology, National Taiwan University, Taipei 106319, Taiwan; 5 Tajen University, Pingtung 907101, Taiwan

**Keywords:** *Lactiplantibacillus brevis*, probiotic, resistance training, muscle mass, strength

## Abstract

In addition to maintaining good exercise and dietary habits, recent studies have shown that probiotics may have potential benefits for muscle mass and strength. It is worth noting that the effects may vary depending on the specific strains used. To date, no studies have analyzed the effects of *Lactiplantibacillus brevis* in this context. Here, we combine the *L. brevis* strain GKEX with resistance training to further understand its effects on muscle mass, thickness, performance, and fat loss. In a six-week intervention for a double-blind randomized trial, 52 healthy subjects were divided into two groups (10 male and 16 female participants in each group): a placebo group (two capsules/day, containing 0 CFU of GKEX per capsule) and a GKEX group (two capsules/day, containing 1 × 10^10^ CFU of GKEX per capsule). Before the intervention, no differences were observed between the two groups in any of the tests (body composition, muscle thickness, exercise performance, and blood parameters). However, supplementation with GKEX significantly improved muscle mass and thickness, as well as grip strength, muscle strength, and explosive performance, when compared to the associated parameters before the intervention. Additionally, GKEX supplementation promoted a reduction in the body fat percentage (*p* < 0.05). Through analysis of the change amount, we observed that GKEX supplementation yielded significantly improved benefits when compared to the placebo group (*p* < 0.05). In summary, our findings support the notion that a six-week resistance exercise training program combined with *L. brevis* GKEX supplementation has superior additive effects that enhance muscle mass and strength performance, while also reducing body fat percentage. This intervention can promote muscle gain and fat loss.

## 1. Introduction

Appropriate and regular exercise training is considered to be effective in preventing injuries, improving muscle mass and strength performance, improving the physiological metabolism, and so on [1]. In addition to exercise training, appropriate nutritional supplementation also plays a considerable role in exercise performance and promoting muscle growth and synthesis [2]. In the past, common sports nutrition supplements were nothing more than antioxidant or protein supplements; however, in recent years, more and more studies have shown that probiotics are supplements with the potential to increase muscle mass and exercise performance. In a past meta-trial combining the results of 24 studies, it was found that probiotic supplementation significantly improved skeletal muscle mass and strength performance, although it did not help total lean mass [3]. In addition, another study has also shown that supplementation with *Lactiplantibacillus plantarum* TWK10 for six consecutive weeks without exercise training significantly improved the muscle mass as well as reducing the body fat rate and exercise fatigue indicators of non-athletes [4].

Probiotics are live micro-organisms that, when taken orally for several weeks and ingested in sufficient amounts, increase the number of beneficial bacteria in the gut, thereby providing health benefits to the host [5,6]. More and more evidence has demonstrated that the gut microbiota can stimulate the IGF-1/p70s6k/mTOR pathway in muscle cells through the synthesis of amino acids, thereby promoting muscle synthesis [7]. The exact mechanism by which probiotics stimulate increases in muscle mass or strength is currently unknown, but the most commonly accepted factor may be through improved protein digestion and amino acid absorption into the bloodstream [8], thereby promoting skeletal muscle anabolism and improving muscle mass and functional performance [9]. Some past studies have confirmed that probiotic supplementation can modulate the gut microbiota to enhance protein assimilation, upregulate mTOR activation and mitochondrial function, and reduce inflammatory cytokine activity, thereby improving muscle anabolism and reducing muscle loss [10,11]. A meta-analysis of the overall effects reported in seven studies has also shown that probiotic supplementation improved muscle mass and strength [12].

*Lactobacillus brevis* is a heterofermentative Gram-positive organism which is widely present in fermented foods of plant and animal origin, highly adhesive to low-pH bile salts and pancreatic juice, competitive against *Salmonella* and *Escherichia coli*, and promotes primary bile acid biosynthesis, amino acids, and other metabolic pathways [13,14]. A previous study has pointed out that daily supplementation with *L. brevis* FZU0713 for 8 weeks significantly regulated liver genes (including Acat2, Acox1, Hmgcr, Cd36, Srebp-1c, and Cyp7a1), thereby promoting the lipid metabolism and maintaining bile acid stability. In addition, they also found that the levels of short-chain fatty acids (SCFAs) in the feces of HFD-fed rats were significantly increased, which are considered to be able to effectively change the composition and metabolic function of gut micro-organisms [15]. Another previous study has shown that SCFAs play an important role in skeletal muscle function and exercise capacity [16], possibly through activating multiple regulatory pathways (e.g., UCP2-AMPK-ACC and PGC1-α) to increase ATP production and muscle fiber metabolic efficiency to affect skeletal muscle performance [17]. In addition, they act by inhibiting histone deacetylase, thereby preventing apoptosis and preventing muscle protein catabolism [18].

While there are no studies directly confirming the relationship between *L*. *brevis* and muscle and exercise performance, past studies have shown that *L*. *brevis* can increase the production of gamma-aminobutyric acid (GABA), the main inhibitory neurotransmitter in the mammalian central nervous system [19]. It can increase plasma growth hormone concentrations, promoting amino acid transport, cellular amino acid uptake, and skeletal muscle growth [20]. A previous study demonstrated that when whey protein was combined with GABA supplementation and resistance exercise training for 12 weeks, it significantly increased growth hormone levels and total body lean tissue weight [21]. Therefore, this study aims to investigate whether supplementation with *L. brevis* combined with resistance exercise training can provide benefits in increasing muscle mass and strength performance, and further explore its adaptability effects on the human body. We hypothesize that the synergistic effect of *L. brevis* combined with resistance exercise training can promote muscle mass and strength performance. This will be the first study to explore the effects of *L*. *brevis* on muscle and exercise performance, serving not only as a foundation for understanding its mechanisms and applications but also as a potential choice for sports nutrition supplementation.

## 2. Materials and Methods

### 2.1. Sample Preparation

*L. brevis* GKEX was isolated from douchi (fermented black soybeans) and preserved at the National Institute of Technology and Evaluation, Biological Resource Center (NBRC, Chiba, Japan), with collection number NITE BP-03696. The bacterial strain was cultured in MRS broth (BD, Franklin Lakes, NJ, USA) for 16 h at 32 °C with agitation at 100 rpm. Subsequently, the seed culture was 0.01% inoculated in a 15T bioreactor containing 80% culture medium as the working volume. The scaled-up culture was maintained at 32 °C for 16 h with pH control set to 6.0. The bioreactor medium comprised 5% glucose, 2% yeast extract, 0.05% MgSO_4_, 0.1% K_2_HPO_4_, and 0.1% Tween 80. Following harvest, *L. brevis* GKEX was centrifuged (MBPX810, Alfa Laval AB, Lund, Sweden) at 7000 rpm and the bacterial pellet was mixed with 10% skim milk for lyophilization (FD24, Kingmech Scientific, Taoyuan, Taiwan) under 30 °C for 5 days. The resulting freeze-dried GKEX powder was then filled into No. 0 vegetable capsules, with each capsule containing 1.0 × 10^10^ CFU of live bacteria. Since the bacterial powder was mixed with microcrystalline α-cellulose and then filled into capsules, we used microcrystalline α-cellulose as a placebo substitute and filled it into the capsules accordingly. All the manufacturing processes were conducted at Grape King Bio Ltd. (Taoyuan, Taiwan) in compliance with the ISO22000 system [22].

### 2.2. Participants

The Harvard calculator (http://hedwig.mgh.harvard.edu/sample_size/size.html, accessed on 20 June 2022) was used to determine the sample size, assuming a parallel design with a significance level of 0.05, a power of 0.8, and a standard deviation of 0.8 for the difference. The trial included a total of 52 subjects (including 20 males and 36 females) from the National Sports University, aged 20–39 years old, who were not athletes. None of them met our exclusion criteria, including (1) BMI ≥ 27; (2) a history of metabolic disease, asthma, cancer, cardiovascular disease, or hypertension; (3) limb and neuromuscular movement disorders occurring within the last 6 months, indicating that the subject is unable to exercise; (4) had taken anti-inflammatory, analgesic, acute, or chronic disease or other drugs in the past month; (5) previous hepatobiliary and gastrointestinal surgery (except hernia and polypectomy); (6) those who smoke or drink; (7) having taken probiotic powder, capsules, or lozenges (including yogurt and other related foods) within two weeks; (8) allergy to food or lactic acid bacteria products; (9) students or stakeholders of the principal investigator. All eligible participants gave written informed consent before starting the experiment. The experiment was performed after receiving approval from the Institutional Review Board of Landseed International Hospital (Taoyuan, Taiwan; LSHIRB No. 22-022-A2) and was registered at clinicaltrials.gov under registration number NCT05909475. This study was conducted according to the guidelines of the Declaration of Helsinki.

### 2.3. Experimental Design

**Figure 1:**
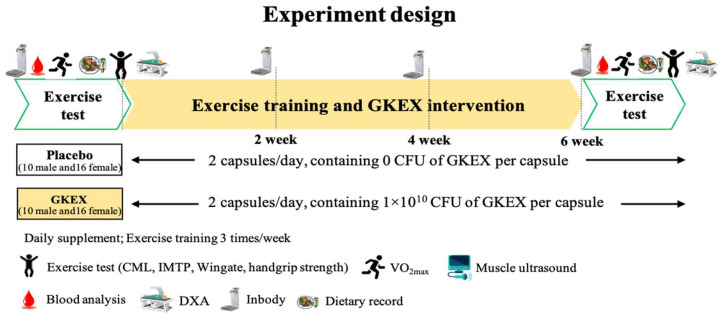
Experimental procedure.

After all subjects were recruited, numbers were assigned according to the order of registration. Then, when the gender ratio of the two groups was the same, we randomly divided them into two groups through numbered sampling and conducted a double-blind experiment (with 10 male and 16 female participants in each group): a placebo group (2 capsules/day, containing 0 CFU of GKEX per capsule) and a GKEX group (2 capsules/day, containing 1 × 10^10^ CFU of GKEX per capsule). There were no significant differences between the placebo and GKEX groups in terms of age (placebo: 22.2 ± 1.9; GKEX: 22.6 ± 2.4 years old) and height (placebo: 166.2 ± 8.9; GKEX: 168.6 ± 8.7 cm). The trial lasted for 6 weeks, during which all subjects were required to supplement with two capsules daily and perform resistance exercise training three times a week. Before and after the intervention, all subjects underwent tests for dietary record, an exercise performance test, and blood samples were collected to analyze and measure body composition indicators. Among these, only body composition was required to be measured every two weeks. Furthermore, all subjects were required to maintain their original lifestyle and habits. The nutrient intake was analyzed by a nutritionist. Table 1 presents the daily nutritional intake of participants, including carbohydrates, proteins, fats, and total calories, both before the experiment and six weeks after the intervention. No notable distinctions were observed between the groups, and there was no statistically significant alteration when comparing the pre- and post-intervention values within each group. The experimental design is shown in Figure 1.

### 2.4. Resistance Exercise Training

All subjects were instructed to engage in resistance training sessions three times per week, utilizing pneumatic resistance training equipment. The equipment comprised abdominal/back 5310, abduction/adduction 3520-HI5, push-up/pull-down 3120, leg extension/curl 3530, twist rehab 5340, and leg press 5540 (AB Hur Oy, Kokkola, Finland). This equipment selection was designed to target the enhancement of strength in specific muscle groups, including the upper limbs, back, abdomen, and legs. The incorporation of pneumatic resistance training equipment provided a standardized platform for participants to engage in targeted exercises, fostering comprehensive strength development in the upper limbs, back, abdomen, and legs. Before initiation of the training regimen, each participant underwent an assessment of their 3RM (repetition maximum) and, subsequently, their 1RM (repetition maximum) was calculated. This calculation involved the application of a coefficient formula derived from the prior literature, ensuring the determination of an appropriate resistance training intensity [23]. The training protocol spanned four weeks, with distinct progressions in intensity. In the initial week, participants executed a set of 15 repetitions at an intensity equivalent to 60% of their 1RM. Progressing to the second week, the intensity was elevated to 70% of 1RM, with a set comprising 12 repetitions. In the subsequent week, there was a further increase in intensity to 75% of 1RM, maintaining a set of 12 repetitions. Until the last week, subjects consistently maintained an intensity of 75% of 1RM, completing 2 sets of 10 repetitions. Researchers adjusted the resistance equipment weight and number of sets based on each subject’s individual 1RM in accordance with the exercise protocol. Subjects were required to complete the designated movements and sets during each training session, with multiple professionals present to monitor the integrity and safety of their movements.

### 2.5. Maximal Oxygen Uptake (VO2max)

Assessment of the maximum oxygen consumption and exercise performance in the subjects involved the utilization of a treadmill (Pulsar, h/p/cosmos, Traunstein, Bavaria, Germany) and an automated breathing analyzer (Vmax 29c, Sensor Medics, Yorba Linda, CA, USA). Following the manufacturer’s instructions, the flow sensor, as well as the O_2_ and CO_2_ sensors, underwent calibration before each test to guarantee accurate measurements. An automated calibration function was employed for the O_2_ and CO_2_ sensors. Heart rate (HR) monitoring was performed using a Polar heart rate device. Following the Bruce protocol, the treadmill was initiated at a speed of 7.2 km/h, progressively increasing by 1.8 km/h every 2 min until subjects reached a point of fatigue, as outlined in the Bruce protocol [24]. The determination of maximum O_2_ consumption occurred when the respiratory exchange rate (VCO_2_/VO_2_, the volume ratio of CO_2_ produced to O_2_) exceeded 1.10 and coincided with reaching the maximum heart rate (maximum HR  =  220 − age). The average of the three highest peaks in VO_2max_ was computed to derive individual VO_2max_ values.

### 2.6. Handgrip Strength Test

Handgrip strength was quantified in kilograms using a Takei digital grip strength meter (T. K. K. 5401, Takei Scientific Instruments Co., Ltd., Kamo, Niigata, Japan). Prior to the formal testing, subjects were instructed to apply minimal force to the gripper as a preliminary measure, in order to ensure their comprehension of the operating procedure and gripping distance. The researchers randomly assigned either the dominant or non-dominant hand as the starting point for the test. During the examination, participants were directed to exert maximal effort while squeezing the gripper with one hand, sustaining the squeeze for a minimum of 5 s. To mitigate the effects of fatigue, subjects alternated hands at 60 s intervals and repeated the test. This exchange method was repeated three times, and the maximum grip strengths of both the dominant and non-dominant hands were individually recorded [25].

### 2.7. Countermovement Jump Assessment (CMJ)

The CMJ test serves as a means to assess the maximal speed, strength, and explosiveness of the lower body. During this evaluation, participants were directed to place their hands on their hips and position their feet on a Kistler force-measuring platform (9260AA, Kistler GmbH, Winterthur, Switzerland). Subsequently, they were instructed to execute a squat until their knees reached a 90-degree bend, followed with an immediate jump with maximal force to attain the highest possible vertical height. Each participant performed three replicates of the CMJ test, and data were acquired at specified points. The instrument was calibrated for each individual’s weight to ensure accuracy [26]. The parameters measured included the rate of force development (RFD), relative peak force, and jump height. These metrics collectively provided insights into the dynamic capabilities of the lower body, reflecting the participant’s ability to generate force and power during vertical jumping movements.

### 2.8. Isometric Mid-Thigh Pull (IMTP)

The isometric mid-thigh pull (IMTP) serves as a force–time diagnostic tool [27]. For this purpose, customized IMTP test equipment and two force plates (type 9287BA, Kistler Instruments AG, Winterthur, Switzerland) were employed. All participants were positioned with their feet at a consistent width and a rod was placed between the thighs, ensuring an upright torso, neutral spine, and knee and hip angles set at 140° to acquaint them with the IMTP test method. A careful evaluation was conducted to confirm the symmetry of the force trace in the practice test and the stability of the weighing cycle. To minimize the risk of measurement errors associated with changes in posture, each test phase involved repeating the specific measurement at 2 min intervals. The recorded parameters encompassed the average relative peak force (N/kg), rate of force development (RFD), and peak rate of force development (pRFD). The collection of these parameters aimed to provide a nuanced understanding of the isometric force production capabilities of the participants and their potential alterations in response to the strenuous exercise regimen.

### 2.9. Wingate Anaerobic Test (WAnT)

Following a standardized warm-up routine, all participants underwent assessment using the classical Wingate Anaerobic Test (WAnT) on a cycloergometer (Monark 894E, Varberg, Sweden) in a 30 s “go all out” ultramax test. The seat height was adjusted to each participant’s satisfaction, and toe clips were utilized to prevent their feet from slipping off the pedals. Prior to the initial test, a 5 min warm-up at approximately 50 W was conducted. Following the warm-up, two 3 s preparation exercises, with a load of 3% of their own body weight, were administered to familiarize the participants with the resistance [28]. The test commenced with the resistance set on the friction belt of the dynamometer. External loading was individually estimated at 5% of body weight. The recorded results included the relative mean power (W/kg), relative peak power (W/kg), and fatigue index (%), which provide insights into the anaerobic power of the participants, showcasing their mean and peak power outputs, as well as their resistance to fatigue during intense, short-duration efforts.

### 2.10. Body Composition

To assess body composition, a multi-frequency approach utilizing a bioelectrical impedance analyzer (BIA) was employed; specifically, an InBody 570 (In-body, Seoul, Republic of Korea) was utilized. This advanced device conducts measurements at frequencies of 1, 5, 50, 260, 500, and 1000 kHz within a 60 s timeframe. During the measurement process, participants stood on the foothold electrodes following clearance of the palms and soles. Holding the sensing handle with both hands, participants maintained an open-arm position at a 30° angle away from their body, refraining from speaking or making movements throughout the measurement period. Additionally, participants observed an 8 h fasting period prior to the tests, ensuring optimal conditions for the assessment of body composition. We conducted measurements every two weeks during the experimental period.

Because BIA is measured in skeletal muscle, it may be affected by the amount of water in the body. Therefore, we additionally used comprehensive and reliable dual-energy X-ray absorptiometry (DXA) measurements before and after the intervention and facilitated the acquisition of additional bone quality data. The Lunar iDXA system from GE Healthcare (Chicago, IL, USA) was used. Subjects were instructed to lie flat on the test bed, aligning their bodies along the centerline with limbs positioned within the detection range to measure the distribution of lean tissue, fat mass, and bone density. The DXA technology involved the use of two X-ray beams with different energies to scan the designated body regions. The scintillation detector received the X-rays that penetrated the inspected parts, facilitating the calculation and analysis of parameters such as muscle mass and body fat.

### 2.11. Ultrasonic Measurement of Muscle and Fascia Thickness

Muscle and fascia thickness measurements of the biceps brachii and quadriceps femoris muscles were conducted using an ultrasonic scanner (H1300, BENQ, Taipei, Taiwan). Participants were positioned lying flat on their backs with palms facing upward, assuming an anatomical position. For the biceps brachii, the measurement position involved determining the straight-line distance from the acromion to the transverse crease of the elbow. The ultrasonic probe was then placed at one-third of the transverse crease of the elbow. Regarding the quadriceps femoris, the muscle probe was positioned from the anterior superior iliac spine (ASIS) to the patella at a one-half straight-line distance from the edge [29]. Muscle thickness analysis was comprised of calculating the average thickness by dividing the sum of the thickness at both ends of the muscle by two. The method for calculating muscle fascia thickness was based on the muscle fascia calculation method [30], defined as muscle thickness × sin(θ), where θ represents the angle between the muscle fascia and the horizontal line. Image analysis was performed using ImageJ version 2.3.0/1.53f (National Institutes of Health, Bethesda, MD, USA).

### 2.12. Clinical Biochemistry and Hematology Analysis

Blood samples were obtained from arm venous catheters both before and after the 6-week intervention as part of the study. The collected samples underwent a comprehensive analysis encompassing liver function, renal function, blood lipids, and glucose levels to assess the metabolic and health status of the subjects. To ensure standardized conditions for blood analysis, all participants were instructed to observe a fasting period of 8 h at minimum prior to blood collection. The blood serum obtained from the samples was then assessed using an autoanalyzer (Hitachi 717, Tokyo, Japan) for various biochemical markers, including aspartate transaminase (AST), aminotransferase (ALT), total cholesterol (TC), triglyceride (TG), high-density lipoprotein cholesterol (HDL-C), low-density lipoprotein cholesterol (LDL-C), blood urea nitrogen (BUN), creatinine (CREA), uric acid (UA), and glucose levels. Complete blood count (CBC) profiles (Sysmex XE-2100, Sysmex Corporation, Kobe, Japan) were also analyzed at the 90 min time point in the recovery phase.

### 2.13. Statistics

All data are expressed as the mean ± SD. Statistical analyses were performed using SPSS Statistics 25 (IBM Co., Armonk, NY, USA). To assess differences within the groups before and after the intervention, a paired Student’s *t*-test was applied for parametric analysis. Post hoc comparisons were analyzed using the Bonferroni test. For non-parametric data, the Wilcoxon signed-rank test was employed. Between-group comparisons were conducted using an unpaired Student’s *t*-test for parametric analyses, while non-parametric data were analyzed using the Mann–Whitney U-test. A significance level of *p* < 0.05 was considered indicative of a statistically significant difference in all analyses.

## 3. Results

### 3.1. Effects of 6-Week Supplementation with GKEX on Body Composition Change

First, Table 2 shows the results of the Inbody body composition measurements every two weeks. We found that, under the same resistance exercise training intervention, there were no significant differences between the placebo and GKEX groups in terms of body weight, BMI, muscle mass, and body fat percentage before the intervention and at 2, 4, and 6 weeks after the intervention. However, through comparison before and after within the groups, it was found that the weight and body fat percentage in the placebo group increased significantly at 2 and 4 weeks after the intervention compared with before the intervention, and the BMI had increased significantly after 4 weeks of intervention (*p* < 0.05). On the other hand, at the 2nd, 4th, and 6th weeks of intervention, compared with before the intervention, the GKEX group presented significantly increased muscle mass (one and one-hundredths-fold, *p* = 0.0005; one and two-hundredths-fold, *p* < 0.0001; and one and three-hundredths-fold, *p* < 0.0001, respectively) and reduced body fat rate (1.76%, *p* = 0.0075; 4.68%, *p* = 0.0001; and 5.23%, *p* = 0.0001, respectively). In addition, increments were calculated separately for weeks 2, 4, and 6 to gain further insight into the changes. The results showed that the placebo group significantly increased in weight, BMI, and body fat percentage compared with the GKEX group at weeks 2, 4, and 6 of the intervention, respectively (*p* < 0.05); meanwhile, the GKEX group had a significantly increased change in muscle mass compared with the placebo group at weeks 2, 4, and 6 of the intervention (*p* < 0.05).

On the other hand, the measurement results from whole-body scans using DXA are shown in Table 3. There was no significant difference between the placebo and GKEX groups in terms of LMB, FBM, and bone weight. However, after 6 weeks of supplementation with GKEX, LBM was significantly increased (by one and one-hundredths-fold, *p* = 0.0231) and FBM was significantly reduced (by 4.88%, *p* = 0.0002) compared to before the intervention. Moreover, regarding the changes in total body fat, the values for the placebo and GKEX groups at 6 weeks were −1.1 ± 1.3 and 0.0 ± 0.9, respectively; notably, the GKEX group presented a significantly reduced total LBM when compared to the placebo group (*p* = 0.0011).

### 3.2. Effects of 6-Week Supplementation with GKEX on Muscle Thickness and Fascia

According to the results shown in Table 4, after 6 weeks of supplementation with GKEX, the right and left quadriceps thicknesses were significantly increased compared to those in the placebo group (by one and nine-hundredths-fold, *p* = 0.0124, and one and nine-hundredths-fold, *p* = 0.0231, respectively). Within-group comparisons revealed that, after 6 weeks of GKEX supplementation, the right biceps were significantly increased (by one and eight-hundredths-fold, *p* < 0.0001), as well as the right quadriceps (by one and five-hundredths-fold, *p* < 0.0001), left biceps (by one and eight-hundredths-fold, *p* < 0.0001), and left quadriceps (by one and three-hundredths-fold, *p* = 0.0020), when compared with those before the intervention. In addition, the change values for the GKEX group were significantly greater than those for the placebo group in the right biceps (*p* < 0.0001), right quadriceps (*p* < 0.0001), left biceps (*p* < 0.0001), and left quadriceps (*p* = 0.0037). There was a significant time effect (*p* < 0.05) and group × time interaction effect (*p* <0.05) for the right biceps, right quadriceps, left biceps, and left quadriceps, respectively. However, no significant differences between groups were detected with the post hoc test.

For the fascia, there was no significant difference between the placebo and GKEX groups regarding the right biceps, right quadriceps, and left biceps, respectively, before and after the intervention. The left quadriceps thickness of the GKEX group was significantly higher than that of the placebo group before the intervention and at 6 weeks after the intervention. In addition, the GKEX group presented significant increases in the right biceps (by 1.20-fold, *p* < 0.0001), right quadriceps (by 1.12-fold, *p* = 0.0092), and left biceps (by 1.09-fold, *p* = 0.0067) compared with before the intervention. The change values in the GKEX group were only significantly greater than those in the placebo group for the right biceps (*p* < 0.0001).

### 3.3. Effects of 6-Week Supplementation with GKEX on Maximum Handgrip Strength

**Figure 2:**
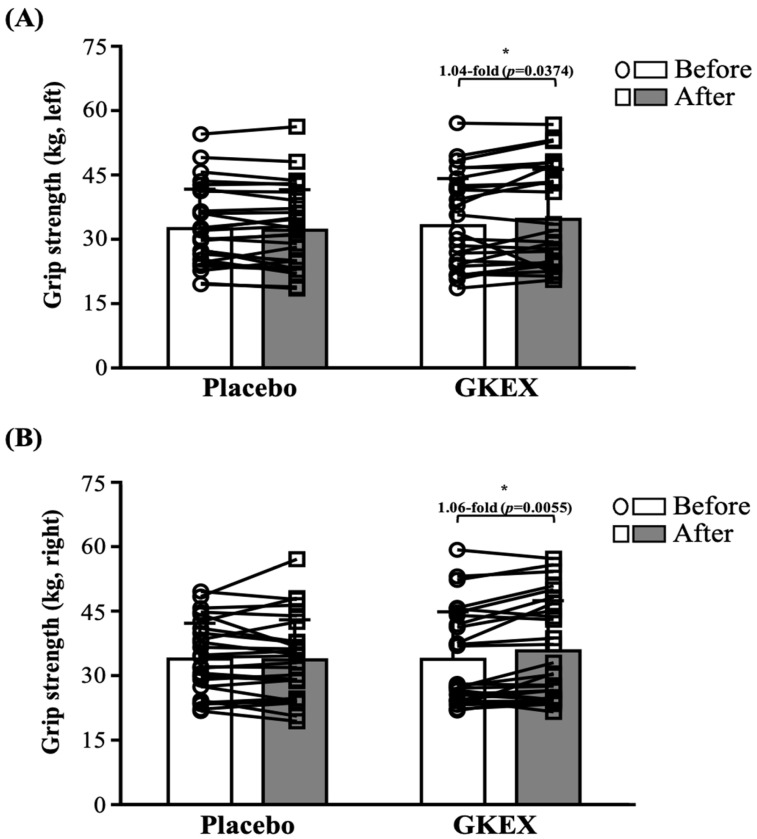
Experimental procedure description. Effects of GKEX combined with resistance training intervention on (**A**) left handgrip strength and (**B**) right handgrip strength. Data are shown as mean ± SD. * indicates significant difference before and after the intervention within a group (*p* < 0.05).

As shown on Figure 2, at both before the intervention and after 6 weeks of intervention, there were no significant differences between the placebo and GKEX groups in terms of left and right maximum handgrip strength. However, in terms of the within-group comparison, only the GKEX supplementation group presented significantly increased right and left maximum handgrip grip strength (by one and four-hundredths-fold, *p* = 0.0374, and one and six-hundredths-fold, *p* = 0.0055, respectively) compared with before the intervention (Figure 2A,B).

### 3.4. Effects of 6-Week Supplementation with GKEX on Maximal Oxygen Uptake (VO_2max_)

**Figure 3:**
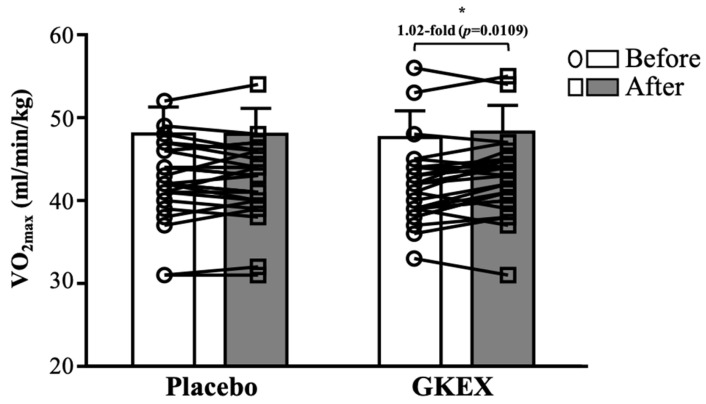
Effects of GKEX combined with resistance training intervention on: VO_2max_. Data are shown as mean ± SD. * indicates significant difference before and after the intervention within a group (*p* < 0.05).

Before the intervention, there were no significant differences in VO_2max_ between the placebo and GKEX groups. After the 6-week intervention, the VO_2max_ values were 42.9 ± 4.9 and 42.5 ± 4.8 (mL/min/kg) in the placebo and GKEX groups, respectively, and there was no significant difference between the two groups. However, within the groups, that in the GKEX group was significantly increased (by one and two-hundredths-fold, *p* = 0.0109) compared to before the intervention (Figure 3). There was a significant time effect (*p* < 0.05) and group × time interaction effect (*p* <0.05) for VO_2max_.

### 3.5. Effects of 6-Week Supplementation with GKEX on Maximum Vertical Jump Height of Exercise Performance

**Figure 4:**
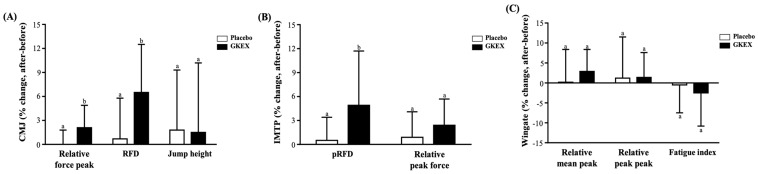
Effects of GKEX combined with resistance training intervention on percentage change in the following: (**A**) CMJ test; (**B**) IMTP test; (**C**) Wingate test. Data are shown as mean ± SD. Different superscript letters (a, b) above the bars indicate significant differences between groups at the same time point (*p* < 0.05). RFD, rate of force development.

Although there were no significant differences in the relative force peak, RFD, and jump height between the placebo and the GKEX groups before and after the intervention, after 6 weeks of supplementation with GKEX, significant increases in the relative force peak (by one and two-hundredths-fold, *p* = 0.0005) and RFD (by one and seven-hundredths-fold, *p* < 0.0001) were observed (Table 5). In addition, through the percentage change, we found that the GKEX group presented a significantly increased relative force peak (*p* = 0.0024; placebo: 2.1 ± 2.8% vs. GKEX: 0.0 ± 1.8%) and RFD (*p* = 0.0004; placebo: 6.5 ± 6.0% vs. GKEX: 0.7 ± 5.1%) (Figure 4A).

### 3.6. Effects of 6-Week Supplementation with GKEX on Isotonic Muscle Strength of Exercise Performance

Table 5 indicates that there were no significant differences in the relative peak force and RFD between the placebo and GKEX groups before and after the intervention; however, after 6 weeks of supplementation with GKEX, significant increases in the relative peak force (by one and five-hundredths-fold, *p* = 0.0032) and RFD (by one and three-hundredths-fold, *p* = 0.0009) were observed. We found that the GKEX group had a significantly increased percentage change in the relative peak force (*p* = 0.0046; placebo: 4.9 ± 6.8% vs. GKEX: 0.5 ± 2.9%) (Figure 4B).

### 3.7. Effects of 6-Week Supplementation with GKEX on Anaerobic Exercise Performance

There were no significant differences in the relative mean power, relative peak power, and fatigue index between the placebo and GKEX groups before and after the intervention. However, after 6 weeks of supplementation with GKEX, there was only a significant increase in the relative mean power (by one and three-hundredths-fold, *p* = 0.0112) (Table 5), and no significant difference in the percentage change was observed (Figure 4C).

### 3.8. Effects of 6-Week GKEX Intervention on Biochemical and Hematological Characteristics of Subjects

All participants underwent blood parameter analysis before and after the intervention to determine their physiological status. This was carried out to ensure that the 6-week GKEX intervention did not result in any adverse side-effects. The study confirmed that all subjects were in good health during the trial, and the intervention did not result in any changes in the various blood indicators measured. No significant changes were observed within each group after the intervention when compared to the pre-intervention period. Moreover, no adverse events related to the intervention were reported during the study (Table 6).

## 4. Discussion

In recent years, a growing body of research has explored the concept of the gut–muscle axis [31], and further evidence has emerged to support the beneficial effects of probiotics in promoting muscle growth and improving exercise performance [32]. However, this study is the first to examine the benefits of supplementation with *L. brevis* to increase muscle mass and exercise performance. In the current study, we found that 6 weeks of supplementation with GKEX and combined with resistance training three times/week could significantly improve muscle strength performance, explosive force, muscle mass, and muscle thickness compared with before the intervention. Supplementation with GKEX combined with resistance exercise training not only effectively increased muscle mass and strength, but it was also found to effectively reduce the body fat.

Resistance training has also been shown to improve the diversity and composition of the gut microbiota. It can increase the richness of SCFA-producing gut microbiota and reduce the relative abundance of pro-inflammatory-inducing species [33]. Previous research has shown that elite rugby players have a higher gut microbial diversity compared to sedentary individuals. Additionally, athletes have a greater proportion of bacteria that are involved in the carbohydrate and amino acid metabolism, resulting in an increased production of SCFAs in the gut, such as acetate, butyrate, and propionate [34]. Among these, butyrate has been shown to prevent cell apoptosis and muscle protein degradation by inhibiting histone deacetylase, and can also increase ATP and muscle fiber metabolic efficiency by activating the UCP2-AMPK-ACC and PGC1-α pathways [18]. In addition, some studies have shown that butyric acid can activate AMPK signaling pathways, stimulate mitochondrial fatty acid oxidation, enhance the glucose metabolism in muscle cells, reduce lipid deposition in muscles, and thereby improve muscle quality and function [18]. It may also promote the proliferation of C2C12 myoblasts by upregulating ERK phosphorylation, in which the expression of myogenic regulatory factors including Myf5 and MyoD are significantly increased, thereby regulating the activation of muscle satellite cells [35]. In one of our previous animal studies, we found that supplementation with *L. plantarum* PL-02 for 4 weeks combined with resistance exercise training significantly increased the muscle mass and strength performance in mice [36]. Moreover, a study in humans without regular exercise habits has also found that supplementation with *L. plantarum* TWK10 for 6 weeks significantly increased muscle mass and endurance performance [4]. Although the *L. brevis* intervention in our study was combined with resistance exercise training, similar results were found in terms of the promotion of muscle mass gain (Table 2 and Table 3). However, we need further research to verify the mechanism by which GKEX promotes muscle growth.

Improvements in muscle strength are thought to be positively correlated with increases in muscle mass [37]. Furthermore, there is also a strong positive correlation between the ability of muscles to produce force and their cross-sectional area (CSA) [38]. Previous research has confirmed that muscle strength is typically determined by the product of muscle mass and the relative strength to muscle volume. Therefore, increasing muscle thickness serves as the basis for achieving strength gains [39]. Despite the use of a muscle ultrasound in our study for measurements, we observed a significant increase in muscle thickness in the GKEX supplementation group compared to before the intervention, which was accompanied by an increase in fascial thickness (Table 4). As greater muscle strength and body mass require thicker connective tissue to withstand and transmit greater forces, increases in muscle thickness may also be accompanied by increases in fascia thickness [40]. This seems to explain our results. In a previous study, continuous supplementation of pea peptides combined with resistance exercise training for 8 weeks significantly enhanced the expression of the insulin-like growth factor 1 receptor and AMP-activated protein kinase. Simultaneously, there was a significant reduction in the expression of myostatin—a muscle growth inhibitor—in the skeletal muscles. This led to an increase in the cross-sectional area of muscles and muscle strength in rats [41]. Although one possible way that probiotics can enhance muscle mass or strength is by improving protein digestion and the absorption of amino acids into the bloodstream [3], the *L. brevis* used in the intervention of this study has not been proven to have this effect. Increased muscle mass is thought to have a positive impact on the rate of strength development, thereby improving movements such as sprinting, jumping, and the ability to change direction [42]. In this study, grip strength, CMJ, IMTP, and anaerobic power tests were performed to assess changes in muscle strength and explosive performance [43]. Although there were no significant differences in various measurements between the two groups, we observed a notable increase in performance only in the group supplemented with GKEX when compared to that before the intervention (Figure 1 and Table 5). Moreover, the GKEX supplementation group exhibited more significant improvements in muscle strength and explosive performance compared to the placebo group, as demonstrated by the magnitude of changes (Figure 4). Previous research in patients with sarcopenia has reported that daily supplementation for 16 weeks with a mixture of various strains totaling 1.12 trillion led to significant improvements in grip strength and gait speed [44]. Another study, considering supplementation with inactivated and coagulated spore-forming bacteria and conducted in soldiers participating in a self-defense course, observed an increase in vertical jump power [45]. These findings support the potential of probiotics in promoting the development of muscle strength. However, further exploration is needed to understand the mechanisms through which *L. brevis* can enhance muscle mass and strength performance.

In addition to influencing muscle mass, probiotics are thought to have the potential to improve endurance performance, although several potential mechanisms remain uncertain. Previous research has suggested that supplementation with a mixture of different probiotics can attenuate the exercise-induced decrease in circulating tryptophan concentrations. This is thought to maintain normal serotonin metabolism, thereby slowing fatigue and preserving exercise performance [46]. Studies have suggested that an increase in training volume and compliance might lead to a reduction in the frequency of upper respiratory tract infections (URTIs). In a trial involving female adolescent swimmers, it has been observed that continuous daily supplementation with 400 mL of probiotic yogurt containing 4 × 10^10^ CFU/mL (including *Lactobacillus acidophilus* SPP, *Lactobacillus delbrueckii Bulgaricus*, *Bifidobacterium bifidum*, and *Streptococcus salivarius subsp. thermophilus*) for 8 weeks led to a significant reduction in the frequency and duration of URTIs, as well as an improvement in maximal oxygen consumption [47]. In our previous study, we found that continuous supplementation with *L. plantarum* TWK10 for six weeks led to a significant improvement in exercise endurance performance in mice. Additionally, there were notable increases in muscle mass and glycogen storage [48]. Glycogen is a polymer of glucose that is related to muscle mass. It is catabolized by glycogen to produce adenosine triphosphate (ATP), which provides the primary fuel source for muscles during exercise [49]. Bacteria in the gut may produce SCFAs, of which butyrate can maintain blood sugar stability and promote liver glucose metabolism through the GPR43-AKT-GSK3 signaling pathway [50]. This can improve exercise endurance performance, delay fatigue, and accelerate recovery [51]. Although it was found that 6 weeks of resistance exercise training combined with GKEX supplementation significantly increased VO_2max_ (Figure 3) in this study, further research is required to gain a deeper understanding of the mechanisms involved.

Although this study confirmed the benefits of *L. brevis* GKEX in terms of improving muscle mass and strength performance, there are limitations and possible mechanisms that require further exploration. To ensure gender equality, no special restrictions were imposed on recruitment, resulting in a slightly different gender ratio between the two groups of subjects. However, the implications of gender differences in the sample and the composition of the sample being comprised of physical education non-majors, specifically, physiological differences between males and females, such as in skeletal muscles, energy metabolism, and hormonal profiles, can lead to larger standard deviations within the group. These differences can potentially affect the statistical results and interpretations of the study [52]. This study primarily investigated the impact of GKEX on enhancing muscle mass and exercise performance, necessitating the inclusion of both male and female subjects in the same group for comparison. Therefore, it may be necessary to include more subjects in future trials to further compare the effects and mechanisms of this sample in both genders. Second, all participants were non-physical education majors from the same university. Although their living conditions were similar and variability was low, this does not necessarily mean that the results apply to the wider ethnic group or population. Third, the original purpose of this study was to explore efficacy and, so, the group design did not compare whether sedentary control or supplementation alone had substantial benefits. Furthermore, apart from this study, no research has been conducted on the correlation between *L. brevis* GKEX and muscle and exercise performance. In summary, the current study demonstrates that *L. brevis* GKEX supplementation combined with resistance exercise training improves muscle mass and strength performance. Although there have been many studies in the past showing the benefits of combining probiotics with exercise training on athletic performance and even competitive athletes [53], this study aimed to investigate its efficacy. To minimize the burden on participants, we collected a limited number of specimens. Once the efficacy test results are confirmed, we plan to delve deeper into the mechanisms using more rigorous experimental designs and analysis projects. Finally, to gain a deeper understanding of the cellular mechanisms underlying our findings, it may be necessary to conduct muscle biopsies or in vitro animal studies. Based on the above, a broader perspective is needed to confirm our findings. We aim to delve deeper into the mechanisms of muscle growth and the role of the gut–muscle axis by analyzing the gut microbiota. We anticipate that, through continued research into these mechanisms and the efficacy results obtained from this study, our findings can be applied to athletes, offering a potential avenue for sports nutrition supplements. Further research in the future needs to develop more stringent experimental design and condition restrictions, and make appropriate adjustments based on the characteristics of the probiotics and the intervention time [54].

## 5. Conclusions

This study demonstrated the effectiveness of combining *L. brevis* GKEX with resistance exercise training for the improvement of muscle mass and strength performance. After six weeks of supplementation and training, GKEX improved muscle mass, thickness, strength, and power. Additionally, it significantly reduced body fat percentage. Further research is needed in the future to elucidate the key mechanism of *L. brevis* GKEX in the gut–muscle axis through in vivo and in vitro studies, and to analyze the relationship between intestinal microbial analysis and muscle synthesis pathways.

## Figures and Tables

**Table 1:** Dietary intake of subjects before and after the 6-week GKEX intervention.

Dietary Intake	Before		After	
	Placebo	GKEX	Placebo	GKEX
Carbohydrates (g/day)	164 ± 38	164 ± 42	163 ± 44	165 ± 43
Proteins (g/day)	124 ± 547	126 ± 39	125 ± 55	125 ± 42
Fats (g/day)	70 ± 26	71 ± 19	70 ± 25	72 ± 19
Total calories (kcal/day)	1785 ± 521	1803 ± 376	1784 ± 522	1805 ± 378

Data are presented as mean ± SD.

**Table 2:** Body composition of subjects, measured every two weeks using Inbody, under the 6-week GKEX combined with resistance training intervention.

Body Composition	Week	Placebo	GKEX	Delta	
				Placebo	GKEX
BW (kg)	Before	61.9 ± 9.2 ^a^	65.3 ± 12.6 ^a^	0.0 ± 0.0 ^a^	0.0 ± 0.0 ^a^
	Week 2	62.2 ± 9.4 ^a,^*	65.0 ± 12.4 ^a^	0.4 ± 0.9 ^b^	−0.3 ± 0.8 ^a^
	Week 4	62.4 ± 9.5 ^a,^*	64.9 ± 12.4 ^a^	0.5 ± 0.8 ^b^	−0.3 ± 1.1 ^a^
	After	62.2 ± 9.5 ^a^	64.7 ± 12.3 ^a^	0.3 ± 0.9 ^b^	−0.5 ± 1.4 ^a^
BMI (kg/m^2^)	Before	22.4 ± 2.7 ^a^	22.8 ± 2.6 ^a^	0.0 ± 0.0 ^a^	0.0 ± 0.0 ^a^
	Week 2	22.5 ± 2.7 ^a^	22.7 ± 2.5 ^a^	0.1 ± 0.3 ^b^	−0.1 ± 0.3 ^a^
	Week 4	22.5 ± 2.7 ^a,^*	22.6 ± 2.5 ^a^	0.2 ± 0.3 ^b^	−0.1 ± 0.4 ^a^
	After	22.5 ± 2.7 ^a^	22.6 ± 2.5 ^a^	0.1 ± 0.3 ^b^	−0.2 ± 0.4 ^a^
Muscle Weight (kg)	Before	27.0 ± 4.9 ^a^	28.6 ± 6.9 ^a^	0.0 ± 0.0 ^a^	0.0 ± 0.0 ^a^
	Week 2	26.9 ± 4.9 ^a^	28.8 ± 6.8 ^a,^*	−0.1 ± 0.4 ^a^	0.3 ± 0.3 ^b^
	Week 4	27.0 ± 5.0 ^a^	29.1 ± 6.8 ^a,^*	0.0 ± 0.3 ^a^	0.5 ± 0.4 ^b^
	After	27.0 ± 5.0 ^a^	29.3 ± 6.9 ^a,^*	0.0 ± 0.5 ^a^	0.8 ± 0.5 ^b^
Fat (%)	Before	21.2 ± 6.9 ^a^	22.4 ± 5.4 ^a^	0.0 ± 0.0 ^a^	0.0 ± 0.0 ^a^
	Week 2	21.4 ± 6.7 ^a,^*	22.1 ± 5.5 ^a,^*	0.2 ± 0.7 ^b^	−0.4 ± 0.7 ^a^
	Week 4	21.4 ± 6.9 ^a,^*	21.4 ± 5.4 ^a,^*	0.2 ± 0.8 ^b^	−1.1 ± 1.2 ^a^
	After	21.1 ± 6.9 ^a^	21.3 ± 5.3 ^a,^*	−0.1 ± 1.0 ^b^	−1.2 ± 1.3 ^a^

Data are presented as mean ± SD. Different superscript letters (a, b) indicate significant differences among groups at the same time point (*p* < 0.05); * indicates a significant effect after the intervention compared to before the intervention (*p* < 0.05). Delta is the value after the intervention minus before the intervention. BW, body weight; BMI, body mass index; LBM, lean body mass; FBM, fat body mass.

**Table 3:** Body composition of subjects before and after the 6-week GKEX combined with resistance training intervention, measured through DXA.

Body Composition	Before		After		Delta	
	Placebo	GKEX	Placebo	GKEX	Placebo	GKEX
LBM (kg)	46.1 ± 7.6 ^a^	47.7 ± 10.2 ^a^	46.4 ± 7.8 ^a^	48.3 ± 10.1 ^a,^*	0.3 ± 0.9 ^a^	0.6 ± 1.2 ^a^
FBM (kg)	21.1 ± 6.9 ^a^	22.4 ± 5.6 ^a^	21.1 ± 6.9 ^a^	21.3 ± 5.3 ^a,^*	−0.2 ± 5.5 ^b^	−1.1 ± 1.3 ^a^
Mineral Weight (kg)	2.5 ± 0.4 ^a^	2.7 ± 0.5 ^a^	2.5 ± 0.4 ^a^	2.7 ± 0.5 ^a^	0.0 ± 0.0 ^a^	0.0 ± 0.0 ^a^

Data are presented as mean ± SD. Different superscript letters (a, b) indicate significant differences among groups at the same time point (*p* < 0.05). * indicates a significant effect after the intervention compared to before the intervention (*p* < 0.05). Delta is the value after the intervention minus before the intervention. LBM, lean body mass; FBM, fat body mass.

**Table 4:** Effects of 6-week GKEX combined with resistance training intervention on muscle and fascia thickness determined by ultrasound.

Muscle Ultrasound	Before		After		Delta	
	Placebo	GKEX	Placebo	GKEX	Placebo	GKEX
Muscle thickness (cm)						
Right biceps	2.19 ± 0.40 ^a^	2.12 ± 0.41^a^	2.21 ± 0.43 ^a,^*	2.29 ± 0.42 ^a,^*	0.02 ± 0.05 ^a^	0.16 ± 0.07 ^b^
Right quadriceps	2.35 ± 0.29 ^a^	2.44 ± 0.30 ^a^	2.35 ± 0.28 ^a^	2.57 ± 0.32 ^a,^*	0.00 ± 0.07 ^a^	0.12 ± 0.07 ^b^
Left biceps	2.13 ± 0.38 ^a^	2.13 ± 0.42 ^a^	1.95 ± 0.37 ^a^	2.29 ± 0.51^a,^*	0.00 ± 0.08 ^a^	0.16 ± 0.14 ^b^
Left quadriceps	2.31 ± 0.26 ^a^	2.44 ± 0.36 ^a^	2.31 ± 0.26 ^a^	2.52 ± 0.37 ^b,^*	0.00 ± 0.06 ^a^	0.08 ± 0.12 ^b^
Fascia (cm)						
Right biceps	0.13 ± 0.07 ^a^	0.11 ± 0.09 ^a^	0.12 ± 0.07 ^a^	0.13 ± 0.09 ^a,^*	0.00 ± 0.02 ^a^	0.02 ± 0.02 ^b^
Right quadriceps	0.15 ± 0.06 ^a^	0.16 ± 0.06 ^a^	0.16 ± 0.06 ^a^	0.18 ± 0.07 ^a,^*	0.01 ± 0.02 ^a^	0.02 ± 0.04 ^a^
Left biceps	0.13 ± 0.06 ^a^	0.15 ± 0.08 ^a^	0.14 ± 0.06 ^a,^*	0.17 ± 0.08 ^b,^*	0.01 ± 0.02 ^a^	0.01 ± 0.02 ^a^
Left quadriceps	0.16 ± 0.06 ^a^	0.19 ± 0.07 ^b^	0.16 ± 0.06 ^a^	0.20 ± 0.06 ^b^	0.01 ± 0.03 ^a^	0.01 ± 0.03 ^a^

Data are presented as mean ± SD. Different superscript letters (a, b) indicate significant differences among groups at the same time point (*p* < 0.05). * indicates a significant effect after the intervention compared to before the intervention (*p* < 0.05). Delta is the value after the intervention minus before the intervention.

**Table 5:** Effects of 6-week GKEX combined with resistance training intervention on the CMJ, IMTP, and Wingate tests.

Exercise Performance	Before		After	
	Placebo	GKEX	Placebo	GKEX
CMJ				
Relative peak force (N/kg)	14.2 ± 2.1 ^a^	14.3 ± 1.9 ^a^	14.2 ± 2.2 ^a^	14.6 ± 1.9 ^a,^*
RFD (N/kg/s)	7.5 ± 1.7 ^a^	7.4 ± 1.6 ^a^	7.5 ± 1.7 ^a^	7.9 ± 1.6 ^a,^*
Jump height (cm)	28.4 ± 6.0 ^a^	29.7 ± 5.4 ^a^	29.1 ± 6.4 ^a^	30.3 ± 5.9 ^a^
IMTP				
Relative peak force (N/kg)	13.5 ± 2.7 ^a^	13.2 ± 3.0 ^a^	13.6 ± 2.7 ^a^	13.9 ± 3.2 ^a,^*
RFD (N/s)	8039 ± 940 ^a^	8057 ± 855 ^a^	8120 ± 976 ^a^	8261 ± 892 ^a,^*
Wingate				
Relative mean power (W/kg)	5.8 ± 1.0 ^a^	5.9 ± 1.0 ^a^	5.8 ± 1.0 ^a^	6.1 ± 1.0 ^a,^*
Relative peak power (W/kg)	8.5 ± 1.9 ^a^	8.9 ± 1.7 ^a^	8.6 ± 1.8 ^a^	9.0 ± 1.6 ^a^
Fatigue index (%)	48.1 ± 7.5 ^a^	46.1 ± 11.1 ^a^	47.9 ± 6.9 ^a^	45.2 ± 11.0 ^a^

Data are presented as mean ± SD. Superscript letter (a) indicates significant differences among groups at the same time point (*p* < 0.05); * indicates a significant effect after the intervention compared to before the intervention (*p* < 0.05). Delta is the value after the intervention minus that before the intervention. CMJ, countermovement jump; IMTP, isometric mid-thigh pull; RFD, rate of force development.

**Table 6:** Blood biochemical parameters and blood count profiles of subjects before and after the GKEX combined with resistance training intervention.

Characteristics	Before		After	
	Placebo	GKEX	Placebo	GKEX
Blood Biochemical Parameters				
AST (U/L)	18 ± 4	18 ± 4	18 ± 3	18 ± 4
ALT (U/L)	17 ± 3	16 ± 4	16 ± 2	15 ± 4
TC (mg/dL)	161 ± 13	162 ± 12	160 ± 13	161 ± 13
TG (mg/dL)	89 ± 17	88 ± 22	88 ± 18	87 ± 17
HDL (mg/dL)	57.4 ± 6.9	56.6 ± 8.1	57.7 ± 7.2	56.9 ± 7.5
LDL (mg/dL)	85.2 ± 14.4	87.8 ± 8.7	85.0 ± 14.3	86.0 ± 10.6
BUN (mg/dL)	15.2 ± 2.0	15.5 ± 2.4	15.0 ± 2.1	15.0 ± 2.9
CREA (mg/dL)	0.98 ± 0.12	0.97 ± 0.12	0.98 ± 0.12	0.97 ± 0.10
UA (mg/dL)	5.2 ± 0.7	5.0 ± 0.6	5.3 ± 0.7	5.0 ± 0.6
TP (g/dL)	7.2 ± 0.3	7.3 ± 0.3	7.2 ± 0.4	7.3 ± 0.3
Glucose (mg/dL)	85 ± 7	86 ± 5	84 ± 7	85 ± 5
Insulin (μU/mL)	10.93 ± 2.24	11.00 ± 3.1	10.81 ± 2.46	10.93 ± 3.10
Lactate (mmol/L)	1.57 ± 0.27	1.64 ± 0.19	1.56 ± 0.29	1.63 ± 0.20
NH_3_ (μmol/L)	56.8 ± 9.9	53.3 ± 9.3	56.7 ± 10.3	53.1 ± 9.6
Complete Blood Count				
WBC (10^3^ cumm)	6.0 ± 0.9	6.5 ± 1.1	6.2 ± 0.9	6.5 ± 1.5
Neutrophils (%)	53.4 ± 7.2	55 ± 6.4	55.4 ± 6.9	53 ± 6.6
Lymphocytes (%)	36.2 ± 6.6	33.8 ± 6.4	34.0 ± 5.9	36.4 ± 6.0
Monocytes (%)	7.5 ± 1.5	7.8 ± 1.7	7.9 ± 1.7	7.3 ± 1.8
Eosinophils (%)	2.3 ± 1.3	2.7 ± 1.5	2.0 ± 1.2	2.5 ± 1.2
Basophils (%)	0.6 ± 0.2	0.6 ± 0.3	0.6 ± 0.3	0.7 ± 0.3
RBC (MIL/cumm)	4.8 ± 0.4	4.7 ± 0.4	4.8 ± 0.4	15.0 ± 2.9
Hemoglobin (gm/dL)	14.1 ± 1.2	14.0 ± 1.2	14.1 ± 1.3	13.9 ± 1.1
Hematocrit (%)	42.1 ± 2.9	41.9 ± 3.7	41.9 ± 3.8	42.2 ± 3.8
M.C.V (fl)	88.0 ± 2.7	89.1 ± 2.5	88.1 ± 2.5	89.1 ± 2.6
M.C.H (pg)	29.7 ± 1.1	29.9 ± 0.9	29.5 ± 1.1	29.8 ± 1.0
M.C.H.C (%)	33.7 ± 0.6	33.4 ± 0.7	33.6 ± 0.7	33.4 ± 0.6
Platelet count (10^3^/Cumm)	273 ± 51	278 ± 51	262 ± 62	273 ± 55

Data are presented as mean ± SD. AST, aspartate aminotransferase; ALT, alanine aminotransferase; BUN, blood urea nitrogen; CREA, creatine; UA, uric acid; TP, total protein; TC, total cholesterol; TG, triacylglycerol; HDL, high-density lipoprotein; LDL, low-density lipoprotein; WBC, white blood cell.

## Data Availability

The original contributions presented in the study are included in the article, further inquiries can be directed to the corresponding author.
